# Explicit Finite Difference Methods for the Delay Pseudoparabolic Equations

**DOI:** 10.1155/2014/497393

**Published:** 2014-02-04

**Authors:** I. Amirali, G. M. Amiraliyev, M. Cakir, E. Cimen

**Affiliations:** ^1^Department of Mathematics, Faculty of Art and Science, Sinop University, 57000 Sinop, Turkey; ^2^Department of Mathematics, Faculty of Science, Yüzüncü Yil University, 65080 Van, Turkey; ^3^Department of Mathematics, Faculty of Education, Yüzüncü Yil University, 65080 Van, Turkey

## Abstract

Finite difference technique is applied to numerical solution of the initial-boundary value problem for the semilinear delay Sobolev or pseudoparabolic equation. By the method of integral identities two-level difference scheme is constructed. For the time integration the implicit rule is being used. Based on the method of energy estimates the fully discrete scheme is shown to be absolutely stable and convergent of order two in space and of order one in time. The error estimates are obtained in the discrete norm. Some numerical results confirming the expected behavior of the method are shown.

## 1. Introduction

We consider the initial-boundary value problem for pseudoparabolic equation with delay in the domain $\bar{Q}=\bar{\Omega }\cup \left[0,T\right]$; $\bar{\Omega }=\left[0,l\right]$, *Q* = *Ω* ∪ (0, *T*], *Ω* = (0, *l*):

(1) Lu≔∂u∂t−∂∂x(a(x,t)∂2u∂t∂x)−∂∂x(b(x,t)∂u∂x)=f(x,t,u(x,t),u(x,t−r)), (x,t)∈Q,u(x,t)=φ(x,t), (x,t)∈Ω¯×[−r,0],u(0,t)=u(l,t)=0, t∈(0,T],

where *r* represents the delay parameter (for simplicity we assume that *T*/*r* is an integer; i.e., *T* = *mr* for some integer *m* > 0), *a*(*x*, *t*) ≥ *α* > 0, |*b*(*x*, *t*)| ≤ b*, and *φ*(*x*, *t*) and *f*(*x*, *t*, *u*_1_, *u*_2_) are given sufficiently smooth functions satisfying certain regularity conditions in $\bar{Q}$ and $\bar{\Omega }\times \left[-r,0\right]$ and $\bar{Q}\times {\mathbb{R}}^{2}$, respectively, to be specified, and furthermore

(2) $$\begin{array}{c} \left|\frac{\partial f}{\partial u_{1}}\right|\leq c^{*}<\infty ,\;\;\left|\frac{\partial f}{\partial u_{2}}\right|\leq d^{*}<\infty . \end{array}$$

Equations of this type arise in many areas of mechanics and physics. Such equations are encountered, for example, as a model for two-phase porous media flows when dynamic effects in the capillary pressure are included [1–3]. They are used also to study heat conduction [4], homogeneous fluid flow in fissured rocks [5], shear in second order fluids [6–8], and other physical models. For a discussion of existence and uniqueness results of pseudoparabolic equations see [1, 9–11]. Various numerical treatments of equations of this type without delay have been considered in [2, 12–19] (see also the references cited in them).

In the present paper finite difference technique is applied to numerical solution of the initial-boundary value problem for the semilinear delay Sobolev or pseudoparabolic equation. By the method of integral identities with use of the piecewise linear basis functions in space and interpolating quadrature rules with weight and remainder term in integral form, two-level difference scheme is constructed (see also [12–14]) for singular perturbation cases without delay. For the time integration we use the implicit rule. The finite difference discretization is shown to be absolutely stable and convergent of order two in space and of order one in time. Based on the method of energy estimates the error analysis for approximate solution is presented. The error estimates are obtained in the discrete norm. Some numerical results confirming the expected behavior of the method are shown.

## 2. Discretization and Mesh

**Notation 2**

Let a set of mesh nodes that discretises *Q* be given by

(3) $$\begin{array}{c} \omega =\omega_{N}\times \omega_{N_{0}}, \end{array}$$

with

(4) $$\begin{array}{c} \omega_{N}=\left\{x_{i}=ih,\;i=1,2,\ldots ,N-1,\;h=\frac{l}{N}\right\},\\ \omega_{N}^{+}=\omega_{N}\cup \left\{x_{N}=l\right\},\;\;{\bar{\omega }}_{N}=\omega_{N}\cup \left\{x_{0}=0,\;x_{N}=l\right\},\\ \omega_{N_{0}}=\left\{t_{j}=j\tau ,\;j=1,2,\ldots ,N_{0},\;\tau =\frac{T}{N_{0}}=\frac{r}{n_{0}}\right\},\\ {\bar{\omega }}_{N_{0}}=\omega_{N_{0}}\cup \left\{t_{0}=0\right\},\\ \omega_{n_{0}^{-}}=\left\{t_{j}=j\tau ,j=-n_{0},\ldots ,0\right\}. \end{array}$$

Define the following finite differences for any mesh function *v*_*i*_ = *v*(*x*_*i*_) given on ${\bar{\omega }}_{N}$ by

(5) $$\begin{array}{c} v_{\bar{x},i}=\frac{v_{i}-v_{i-1}}{h},\;\;v_{x,i}=\frac{v_{i+1}-v_{i}}{h},\\ v_{\overset{{}^{\circ}}{x},i}=\frac{v_{i+1}-v_{i-1}}{2h},\\ v_{\bar{x}x,i}=\frac{v_{x,i}-v_{\bar{x},i}}{h}=\frac{v_{i+1}-2v_{i}+v_{i-1}}{h^{2}}. \end{array}$$

Introduce the inner products for the mesh functions *v*_*i*_ and *w*_*i*_ defined on ${\bar{\omega }}_{N}$ as follows:

(6) $$\begin{array}{c} {\left(v,w\right)}_{0}\equiv {\left(v,w\right)}_{\omega_{N}}≔\overset{N-1}{\underset{i=1}{\sum }}hv_{i}w_{i},\\ \left(v,w\right]\equiv {\left(v,w\right)}_{\omega_{N}^{+}}≔\overset{N}{\underset{i=1}{\sum }}hv_{i}w_{i}. \end{array}$$

For any mesh function *v*_*i*_, vanishing for *i* = 0 and *i* = *N* we introduce the norms

(7) $$\begin{array}{c} {||v||}_{0}^{2}\equiv {||v||}_{0,\omega_{N}}^{2}≔{\left(v,v\right)}_{0},\\ {||v_{\bar{x}}||}_{0}^{2}\equiv {||v_{\bar{x}}||}_{0,\omega_{N}^{+}}^{2}≔\left(v_{\bar{x}},v_{\bar{x}}\right],\\ {||v||}_{1}^{2}={||v||}_{0}^{2}+{||v_{\bar{x}}||}_{0}^{2},\\ {||v||}_{\infty }\equiv {||v||}_{\infty ,\omega_{N}}≔\underset{1\leq i\leq N-1}{\max}\left|v_{i}\right|, \end{array}$$

and “negative” norm for any function *w*_*i*_ (1 ≤ *i* ≤ *N* − 1)

(8) $$\begin{array}{c} {||w||}_{-1}≔\underset{v≢0}{\sup}\frac{\left|{\left(w,v\right)}_{0}\right|}{{||v||}_{1}}. \end{array}$$

Given a function $g\equiv g_{i}^{j}\equiv g(x_{i},t_{j});\;\overset{˘}{g}=g_{i}^{\left(j-1\right)}$, defined on $\bar{\omega }$, we will also use the notation

(9) $$\begin{array}{c} g_{\bar{t},i}^{j}=\frac{g_{i}^{j}-g_{i}^{j-1}}{\tau_{j}},\;\;g_{t,i}^{j}=\frac{g_{i}^{j+1}-g_{i}^{j}}{\tau_{j+1}}. \end{array}$$

### 2.1. Difference Scheme

The approach of generating difference scheme is through the integral identity

(10) τ−1h−1∫tj−1tj∫xi−1xi+1Luψi(x) dx dt  =τ−1h−1∫tj−1tj∫xi−1xi+1f(x,t,u(x,t),u(x,t−r))         ×ψi(x) dx dt,

with the usual piecewise linear basis functions for the space

(11) $$\begin{array}{c} \psi_{i}\left(x\right)=\{\begin{array}{cc} h^{-1}\left(x-x_{i-1}\right), & x_{i-1}<x<x_{i},\\ h^{-1}\left(x_{i+1}-x\right), & x_{i}<x<x_{i+1},\\ 0, & x\notin \left(x_{i-1},x_{i+1}\right),\\ & i=1,2,\ldots ,N-1. \end{array} \end{array}$$

Using the appropriate interpolating quadrature rules with weight and remainder term in integral form, consistent with [12–14], we obtain the precise relation

(12) ℓu≔ut¯−(Aut¯x¯)x−(Bux¯)x+R=f(x,t−τ,u(x,t−τ),u(x,t−τ−r)),(x,t)∈ωN×ωN0,

where

(13) $$\begin{array}{c} A=\tau^{-1}h^{-1}\overset{t_{j}}{\underset{t_{j-1}}{\int }}\overset{x_{i}}{\underset{x_{i-1}}{\int }}a\left(x,t\right)\;dx\;dt,\\ B=\tau^{-1}h^{-1}\overset{t_{j}}{\underset{t_{j-1}}{\int }}\overset{x_{i}}{\underset{x_{i-1}}{\int }}b\left(x,t\right)\;dx\;dt. \end{array}$$

The remainder term *R* has the form

(14) $$\begin{array}{c} R={\left(R^{\left(0\right)}\right)}_{x}+R^{\left(1\right)},\;\text{for}\;\left(x,t\right)\in \omega_{N}\times \omega_{N_{0}} \end{array}$$

with

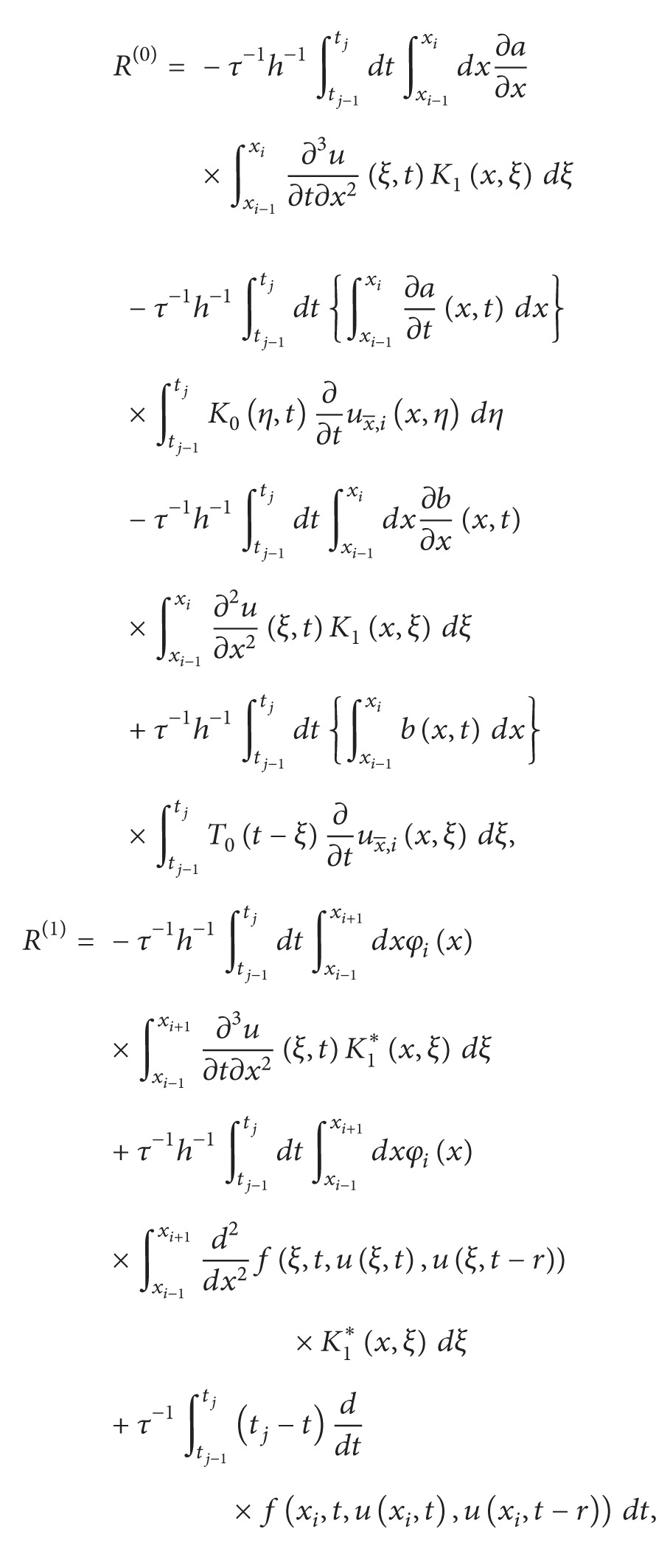
(15)

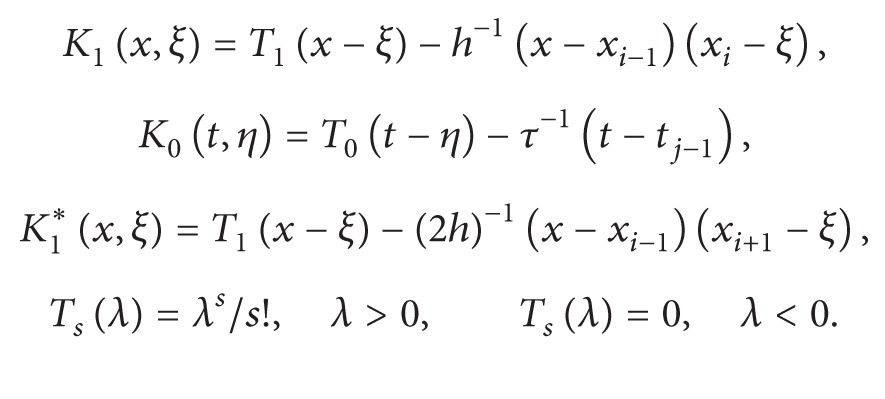
(16)
Based on (12), we propose the following difference scheme for approximating (1):

(17) ℓy=f(x,t−τ,y(x,t−τ),y(x,t−τ−r)),(x,t)∈ωN×ωN0,y(0,t)=y(l,t)=0, t∈ω¯N0,y(x,0)=φ(x,0), x∈ω¯N,

where *ℓy* is defined by (12).

## 3. The Error Estimates and Convergence

To estimate the convergence of this method, note that the error function *z* = *y* − *u* is the solution of the discrete problem,

(18) ℓz=f(x,t−τ,y(x,t−τ),y(x,t−τ−r))−f(x,t−τ,u(x,t−τ),u(x,t−τ−r))+R,(x,t)∈ωN×ωN0,z(x,t)=0, (x,t)∈ω¯N×ωn0−,z(0,t)=z(l,t)=0, t∈ωN0.

Before obtaining the estimate for the solution (18) we give the following Lemma.

**Lemma**

Let the mesh function *δ* ≥ 0, defined on *ω*_*N*_0__, satisfy

(19) $$\begin{array}{c} \delta_{j}\leq \alpha +\tau \overset{j}{\underset{k=1}{\sum }}\left\{c_{1}\delta_{\text{k}-1}+c_{*}\delta_{j-n_{0}-1}+\rho_{k}\right\},\;j=1,2,\ldots ,N_{0},\\ \delta_{j}\leq \varphi_{j},\;j=-n_{0},\ldots ,0,\;\;\varphi_{0}\leq \alpha , \end{array}$$

where 0 ≤ *α*,  *c*_1_, *c*_∗_ = *const*, *ρ*_*j*_ ≥ 0, *φ*_*j*_ given, *n*_0_ ≥ 0 is an integer. Then

(20) $$\begin{array}{c} \delta_{j}\leq \left(\alpha +c_{*}{||\varphi ||}_{L_{1}(\omega_{n_{0}^{-}})}\right)e^{c_{1}t_{j}}+\tau \overset{j}{\underset{k=1}{\sum }}e^{(c_{1}+c_{*})t_{j-k}}\rho_{k}, \end{array}$$

where

(21) $$\begin{array}{c} {||\varphi ||}_{L_{1}(\omega_{n_{0}^{-}})}=\overset{0}{\underset{j=-n_{0}}{\sum }}\tau \varphi_{j}. \end{array}$$

**Proof**

For 1 ≤ *j* ≤ *n*_0_ + 1, − *n*_0_ ≤ *k* − 1 − *n*_0_≤0 and inequality (19) reduces to

(22) $$\begin{array}{c} \delta_{j}\leq \alpha +\tau \overset{j}{\underset{k=1}{\sum }}\left(c_{1}\delta_{k-1}+\rho_{k}\right)+c_{*}{||\varphi ||}_{L_{1}(\omega_{n_{0}^{-}})}. \end{array}$$

Applying now the difference analogue of the Gronwall's inequality we get

(23) δj≤(α+c∗||φ||L1(ωn0−))ec1tj+τ∑k=1jec1tj−kρk,1≤j≤n0+1.

For *j* > *n*_0_ + 1, after replacing in (19) *k* − *n*_0_ = *p*, we have

(24) δj≤α+τ∑k=1j{c1δk−1+ρk}+τ∑p=1−n0j−n0c∗δk−1=α+τ∑k=1j{c1δk−1+ρk}+τ∑p=1−n01c∗δk−1+τ∑p=2j−n0c∗δk−1≤α+τ∑k=1j{(c1+c∗)δk−1+ρk}+c∗||φ||L1(ωn0−),

which by virtue of difference analogue of the Gronwall's inequality leads to (20), immediately.

**Theorem 2**

Let the derivatives ∂*f*/∂*t*, ∂^*s*^ *f*/∂*x* ^*s*^, ∂^1+*k*^ *f*/∂*u*_*s*_∂*x* ^*k*^  (*s* = 1,2; *k* = 0,1),  * and*  ∂^2^ *f*/∂*u*_1_^*k*^∂*u*_2_^2−*k*^  (*k* = 0,1, 2) be continuous and bounded on $\bar{Q}\times {\mathbb{R}}^{2},a$, $b\in C\left(\bar{Q}\right),\left(\partial^{2}/\partial x^{2}\right)\varphi \left(x,t\right),\;(\partial /\partial t)\varphi (x,t)\in C\left(\bar{\Omega }\times \left[-r,0\right]\right)$, and ∂*u*/∂*t*, ∂*u*/∂*x*, ∂^2^ *u*/∂*x* ^2^,  *and* $\partial^{3}u/\partial x^{2}\partial t\in C\left(\bar{Q}\right)$. Then for the discrete problem (17) the following error estimate holds:

(25) $$\begin{array}{c} {||y-u||}_{1}\leq C\left(h^{2}+\tau \right),\;t\in \omega_{N_{0}}. \end{array}$$

**Proof**

Consider identity

(26) (ℓz,zt¯)ωN=(f(x,t−τ,y(x,t−τ−n0τ)) −f(x,t−τ,u(x,t−τ−n0τ)),zt¯)ωN+(R,zt¯)ωN.

After some manipulations, we get

(27) ||zt¯||02+α||zt¯x¯||02≤b∗||z˘x¯||0||zt¯x¯||0+c∗||zt¯||0||z˘||0+d∗||zt¯||0||z(t−τ−n0τ)||0+||zt¯x¯||0||R||−1,12||zt¯||02+α2||zt¯x¯||02≤α−1(b∗)2||z˘x¯||02+(c∗)2||z˘||02+(d∗)2||z(t−τ−n0τ)||02+α−1||R||−12.

Multiplying this inequality by *τ* and summing it up from *k* = 1 to *k* = *j*, also, using here the inequality

(28) $$\begin{array}{c} v_{j}^{2}\leq t_{j}\tau \overset{j}{\underset{k=1}{\sum }}v_{\bar{t},k}^{2}\leq T\tau \overset{j}{\underset{k=1}{\sum }}v_{\bar{t},k}^{2},\;\left(v_{0}=0\right), \end{array}$$

we obtain

(29) ||zj||02+α||zx¯j||02  ≤τ∑k=1j{2α−1T(b∗)2||z˘x¯||02+2(c∗)2T||z˘||02      +2(d∗)2T||z˘(t−r)||02+2Tα−1||R||−12}.

Denoting

(30) $$\begin{array}{c} \delta_{j}={||z^{j}||}_{0}^{2}+\alpha {||z_{\bar{x}}^{j}||}_{0}^{2}, \end{array}$$

we have

(31) $$\begin{array}{c} \delta_{j}\leq \tau \overset{j}{\underset{k=1}{\sum }}\left\{c_{1}\delta_{k-1}+c_{*}\delta_{k-n_{0}-1}+\rho_{k}\right\},\;j⩾1, \end{array}$$

where

(32) $$\begin{array}{c} c_{1}=2T\max\left\{\alpha^{-1}{\left(b^{*}\right)}^{2},{\left(c^{*}\right)}^{2}\right\},\;\;c_{*}=2T{\left(d^{*}\right)}^{2},\\ \rho =2T\alpha^{-1}{||R||}_{-1}^{2}. \end{array}$$

Applying now Lemma 1 we obtain

(33) $$\begin{array}{c} {||z^{j}||}_{0}^{2}+\alpha {||z_{\bar{x}}^{j}||}_{0}^{2}⩽2T\alpha^{-1}\tau \overset{j}{\underset{k=1}{\sum }}e^{(c_{1}+c_{*})t_{k-j}}{||R^{k}||}_{-1}^{2}. \end{array}$$

Further, in view of the fact that

(34) |(R,z)0|≤|(Rx(0),z)0|+|(R(1),z)0|=|(R(0),zx¯]|+|(R(1),z)0|, t∈ωN0,

we obtain

(35) $$\begin{array}{c} {||R||}_{-1}\leq {||R^{\left(0\right)}||}_{0,\omega_{N}^{+}}+{||R^{\left(1\right)}||}_{0,\omega_{N}},\;t\in \omega_{N_{0}}, \end{array}$$

where *R* ^(0)^ and *R* ^(1)^are given by (15). From (35), under the assumed smoothness, we have

(36) $$\begin{array}{c} \tau \overset{N_{0}}{\underset{k=1}{\sum }}{||R^{k}||}_{-1}^{2}=O\left(h^{2}+\tau \right), \end{array}$$

which together with (33) completes the proof of the theorem.

**Remark 3**

Under sufficiently smoothness of *a*(*x*, *t*) and *b*(*x*, *t*) for calculations of   *A* and *B* appropriate numerical quadrature formulae can be applied; for example, *A* = *a*(*x*_*i*_ − *h*/2, *t*_*j*_) , *A* = (1/2)[*a*(*x*_*i*_, *t*_*j*_) + *a*(*x*_*i*−1_, *t*_*j*_)], and so forth.

## 4. Numerical Results

In this section, we present numerical results obtained by applying the numerical method (17) to the particular problems.

**Example**

Consider the following linear problems:

(37) ∂u∂t−2∂3u∂t∂x2−∂2u∂x2+u(x,t−1)=f(x,t),         (x,t)∈[0,1]×[0,2]u(x,t)=e−t(sinh⁡(x)−xsinh⁡(1)),     (x,t)∈[0,1]×[−1,0]u(0,t)=0,  u(1,t)=0, t∈(0,2],

where

(38) $$\begin{array}{c} f\left(x,t\right)=e^{-t}x\sinh\left(1\right)+e^{1-t}\left(\sinh\left(x\right)-x\sinh\left(1\right)\right). \end{array}$$

The exact solution of this problem is *u*(*x*, *t*) = *e* ^−*t*^(sinh⁡(*x*) − *x*sinh⁡(1)). The computational results are presented in Tables 1 and 2.

**Example 2**

Now consider the following nonlinear problem:

(39) ∂u∂t−3∂3u∂t∂x2−2∂2u∂x2+tanh(u(x,t−1))=f(x,t),(x,t)∈[0,1]×[0,2],u(x,t)=e−t(cosh⁡(x)−xcosh⁡(1)+x−1),(x,t)∈[0,1]×[−1,0],u(0,t)=0,  u(1,t)=0, t∈(0,2],

where

(40) f(x,t)=e−t(xcosh⁡(1)−x+1)+tanh(e1−t(cosh⁡⁡(x)−xcosh⁡(1)+x−1)).

The exact solution of this problem is *u*(*x*, *t*) = *e* ^−*t*^(cosh⁡(*x*) − *x*cosh⁡(1) + *x* − 1). The computational results are presented in Tables 3 and 4.

It can be observed that the obtained results are essentially in agreement with the theoretical analysis described above.

## 5. Conclusion

In this paper, we proposed an efficient numerical method for solving initial-boundary value problem for the semilinear pseudoparabolic equation. The proposed finite difference method was constructed, and based on the method of energy estimates the fully discrete scheme was shown to be absolutely stable and convergent of order two in space and of order one in time. The error estimates were obtained in discrete norm. Numerical results were presented, which numerically validate this theoretical result.

## Figures and Tables

**Table 1:** The numerical results on (0,1) × (0,1).

Nodes (*x*, *t*)	Exact solution	Numerical solution *h* = 0.02 *τ* = 0.02	Pointwise error |*y* − *u*|	Numerical solution *h* = 0.02 *τ* = 0.01	Pointwise error |*y* − *u*|
(0.1,0.1)	− 1.570*E* − 02	− 1.571*E* − 02	1.602*E* − 05	− 1.571*E* − 02	8.060*E* − 06
(0.2,0.2)	− 2.759*E* − 02	− 2.765*E* − 02	5.784*E* − 05	− 2.762*E* − 02	2.908*E* − 05
(0.3,0.3)	− 3.558*E* − 02	− 3.570*E* − 02	1.150*E* − 04	− 3.564*E* − 02	5.779*E* − 05
(0.4,0.4)	− 3.976*E* − 02	− 3.994*E* − 02	1.760*E* − 04	− 3.985*E* − 02	8.847*E* − 05
(0.5,0.5)	− 4.033*E* − 02	− 4.056*E* − 02	2.293*E* − 04	− 4.045*E* − 02	1.152*E* − 04
(0.6,0.6)	− 3.757*E* − 02	− 3.783*E* − 02	2.633*E* − 04	− 3.770*E* − 02	1.324*E* − 04
(0.7,0.7)	− 3.180*E* − 02	− 3.207*E* − 02	2.673*E* − 04	− 3.194*E* − 02	1.343*E* − 04
(0.8,0.8)	− 2.338*E* − 02	− 2.362*E* − 02	2.308*E* − 04	− 2.350*E* − 02	1.160*E* − 04
(0.9,0.9)	− 1.267*E* − 02	− 1.281*E* − 02	1.445*E* − 04	− 1.274*E* − 02	7.261*E* − 05

**Table 2:** The numerical results on (0,1) × (1,2).

Nodes (*x*, *t*)	Exact solution	Numerical solution *h* = 0.02 *τ* = 0.02	Pointwise error |*y* − *u*|	Numerical solution *h* = 0.02 *τ* = 0.01	Pointwise error |*y* − *u*|
(0.1,1.1)	− 5.776*E* − 03	− 5.862*E* − 03	8.565*E* − 05	− 5.819*E* − 03	4.203*E* − 05
(0.2,1.2)	− 1.015*E* − 02	− 1.032*E* − 02	1.688*E* − 04	− 1.023*E* − 02	8.481*E* − 05
(0.3,1.3)	− 1.309*E* − 02	− 1.333*E* − 02	2.422*E* − 04	− 1.321*E* − 02	1.217*E* − 04
(0.4,1.4)	− 1.463*E* − 02	− 1.492*E* − 02	2.993*E* − 04	− 1.478*E* − 02	1.504*E* − 04
(0.5,1.5)	− 1.483*E* − 02	− 1.517*E* − 02	3.339*E* − 04	− 1.500*E* − 02	1.678*E* − 04
(0.6,1.6)	− 1.382*E* − 02	− 1.416*E* − 02	3.406*E* − 04	− 1.399*E* − 02	1.711*E* − 04
(0.7,1.7)	− 1.170*E* − 02	− 1.201*E* − 02	3.145*E* − 04	− 1.186*E* − 02	1.580*E* − 04
(0.8,1.8)	− 8.604*E* − 03	− 8.856*E* − 03	2.514*E* − 04	− 8.730*E* − 03	1.263*E* − 04
(0.9,1.9)	− 4.661*E* − 03	− 4.808*E* − 03	1.476*E* − 04	− 4.735*E* − 03	7.415*E* − 05

**Table 3:** The numerical results on (0,1) × (0,1).

Nodes (*x*, *t*)	Exact solution	Numerical solution *h* = 0.02 *τ* = 0.02	Pointwise error |*y* − *u*|	Numerical solution *h* = 0.02 *τ* = 0.01	Pointwise error |*y* − *u*|
(0.1,0.1)	− 4.461*E* − 02	− 4.465*E* − 02	4.503*E* − 05	− 4.463*E* − 02	2.265*E* − 05
(0.2,0.2)	− 7.249*E* − 02	− 7.264*E* − 02	1.490*E* − 04	− 7.257*E* − 02	7.494*E* − 05
(0.3,0.3)	− 8.710*E* − 02	− 8.738*E* − 02	2.736*E* − 04	− 8.724*E* − 02	1.376*E* − 04
(0.4,0.4)	− 9.127*E* − 02	− 9.166*E* − 02	3.893*E* − 04	− 9.146*E* − 02	1.957*E* − 04
(0.5,0.5)	− 8.728*E* − 02	− 8.776*E* − 02	4.741*E* − 04	− 8.752*E* − 02	2.383*E* − 04
(0.6,0.6)	− 7.704*E* − 02	− 7.755*E* − 02	5.115*E* − 04	− 7.730*E* − 02	2.571*E* − 04
(0.7,0.7)	− 6.206*E* − 02	− 6.255*E* − 02	4.896*E* − 04	− 6.231*E* − 02	2.461*E* − 04
(0.8,0.8)	− 4.359*E* − 02	− 4.399*E* − 02	4.003*E* − 04	− 4.379*E* − 02	2.012*E* − 04
(0.9,0.9)	− 2.264*E* − 02	− 2.287*E* − 02	2.381*E* − 04	− 2.276*E* − 02	1.197*E* − 04

**Table 4:** The numerical results on (0,1) × (1,2).

Nodes (*x*, *t*)	Exact solution	Numerical solution *h* = 0.02 *τ* = 0.02	Pointwise error |*y* − *u*|	Numerical solution *h* = 0.02 *τ* = 0.01	Pointwise error |*y* − *u*|
(0.1,1.1)	− 1.641*E* − 02	− 1.663*E* − 02	2.192*E* − 04	− 1.652*E* − 02	1.101*E* − 04
(0.2,1.2)	− 2.667*E* − 02	− 2.706*E* − 02	3.961*E* − 04	− 2.686*E* − 02	1.990*E* − 04
(0.3,1.3)	− 3.204*E* − 02	− 3.257*E* − 02	5.251*E* − 04	− 3.230*E* − 02	2.637*E* − 04
(0.4,1.4)	− 3.357*E* − 02	− 3.417*E* − 02	6.032*E* − 04	− 3.387*E* − 02	3.029*E* − 04
(0.5,1.5)	− 3.211*E* − 02	− 3.274*E* − 02	6.289*E* − 04	− 3.242*E* − 02	3.158*E* − 04
(0.6,1.6)	− 2.834*E* − 02	− 2.894*E* − 02	6.024*E* − 04	− 2.864*E* − 02	3.024*E* − 04
(0.7,1.7)	− 2.283*E* − 02	− 2.335*E* − 02	5.244*E* − 04	− 2.309*E* − 02	2.633*E* − 04
(0.8,1.8)	− 1.603*E* − 02	− 1.643*E* − 02	3.966*E* − 04	− 1.623*E* − 02	1.991*E* − 04
(0.9,1.9)	− 8.328*E* − 03	− 8.550*E* − 03	2.211*E* − 04	− 8.439*E* − 03	1.110*E* − 04
